# Unconscious Imagination and the Mental Imagery Debate

**DOI:** 10.3389/fpsyg.2017.00799

**Published:** 2017-05-23

**Authors:** Berit Brogaard, Dimitria Electra Gatzia

**Affiliations:** ^1^The Brogaard Lab for Multisensory Research, University of Miami, MiamiFL, United States; ^2^Department of Philosophy, University of OsloOslo, Norway; ^3^Department of Philosophy, University of Akron Wayne College, AkronOH, United States; ^4^Centre for Philosophical Psychology, University of AntwerpAntwerp, Belgium

**Keywords:** double dissociation, imagery debate, impoverished phenomenology, type 2 blindsight, unconscious imagination, vision for action, visual imagery

## Abstract

Traditionally, philosophers have appealed to the phenomenological similarity between visual experience and visual imagery to support the hypothesis that there is significant overlap between the perceptual and imaginative domains. The current evidence, however, is inconclusive: while evidence from transcranial brain stimulation seems to support this conclusion, neurophysiological evidence from brain lesion studies (e.g., from patients with brain lesions resulting in a loss of mental imagery but not a corresponding loss of perception and vice versa) indicates that there are functional and anatomical dissociations between mental imagery and perception. Assuming that the mental imagery and perception do not overlap, at least, to the extent traditionally assumed, then the question arises as to what exactly mental imagery is and whether it parallels perception by proceeding via several functionally distinct mechanisms. In this review, we argue that even though there may not be a shared mechanism underlying vision for perception and conscious imagery, there is an overlap between the mechanisms underlying vision for action and unconscious visual imagery. On the basis of these findings, we propose a modification of Kosslyn’s model of imagery that accommodates unconscious imagination and explore possible explanations of the quasi-pictorial phenomenology of conscious visual imagery in light of the fact that its underlying neural substrates and mechanisms typically are distinct from those of visual experience.

## Introduction

It has been hypothesized that there is a significant overlap between the perceptual and the imagistic domains^1^. Early modern philosophers such as Hume (1739/1978) appealed to the phenomenological similarity between visual experience and visual imagery in support of this hypothesis. Centuries later psychologists have appealed to our ability to manipulate mental images in ways similar to the ways in which we would manipulate real objects as a way to provide evidential support for it (see e.g., Shepard and Metzler, 1971; Kosslyn, 1980). If the hypothesis is correct, then we should expect visual mental imagery to be parallel to visual perception in various respects. We know, for example, that visual detection abilities do not require conscious vision (for reviews see, e.g., Brogaard, 2011a,b), so we should expect imaginative abilities to be executable at a level below conscious awareness as well.

Research on brain lesions affecting visual perception, by contrast, do not corroborate the hypothesis that there is a significant functional overlap between perception and mental imagery (for reviews see Farah, 1988; Bartolomeo, 2002). In fact, the current evidence suggests that perception and mental imagery may be functionally and anatomically dissociated. For example, empirical studies indicate that brain lesions that have impaired the ability to perceptually recognize objects or their attributes have left the ability to imagine these types of objects or attributes fairly intact (Guariglia et al., 1993; Chatterjee and Southwood, 1995; Shuren et al., 1996; Beschin et al., 1997; Coslett, 1997; Bartolomeo et al., 1998; Bridge et al., 2012). Similarly, brain injuries that resulted in a loss of imaginative abilities but spared perceptual abilities have been well-documented (Charcot and Bernard, 1883; De Vreese, 1991; Sirigu and Duhamel, 2001; Moro et al., 2008). The overall evidence for double dissociation between perception and imagination suggests that the perceptual and the imagistic domains may not overlap to the extent traditionally assumed – although, as we shall see, evidence from transcranial brain stimulation studies suggest a closer connection between perception and mental imagery.

Assuming that the mechanisms and neural substrates of visual perception and visual mental imagery do not overlap to a significant degree or in the relevant ways, the question arises as to what exactly mental imagery is and whether it parallels perception by proceeding via several functionally distinct mechanisms. In this review paper, we will argue that even though there may not be a shared mechanism underlying vision for perception and conscious imagery, there is a considerable overlap between the mechanisms and neural substrates underlying vision for action and imagined perspective-taking, imagined motion, and imagined rotation. We further argue that these findings provide support for a modification of Kosslyn’s imagery model that takes account of unconscious imagination. We conclude by looking at some possible explanations of the quasi-pictorial phenomenology of conscious visual imagery in light of the fact that its underlying neural substrates and mechanisms are distinct from those of conscious visual perception.

## What is Visual Imagery?

Mental imagery, as we shall use the term, can take a variety of forms. Maintaining imagistic working memory, retrieving episodic memories, daydreaming, visualizing written narratives, imagining a character in pretense games, and modal and counterfactual thinking involve propositional imagery, which is characterized by a ‘that’-clause. For example, you may daydream that you are ice-skating in Antwerp. Or you may imagine a counterfactual scenario in which it is sunny and warm in Antwerp right now. Although mental images are frequently involved in propositional imagery, this form of imagery involves more than merely forming a particular mental image. It involves having a propositional attitude with a particular content (Currie and Ichino, 2013; Van Leeuwen, 2016). Whether such propositional attitudes are belief-like, desire-like, or entirely distinct from belief or desires (e.g., alief, see Gendler, 2008) is a matter of fierce debate (Currie, 2002; Currie and Ichino, 2012). The debate about the nature of propositional imagery extends to imagistic content. For example, it has been argued that such attitudes “inherit” their content from beliefs or desires (Currie and Ravenscroft, 2002, pp. 18–19; Van Leeuwen, 2014, p. 704).

Imaging an object, a taste, a texture, a sound, or an odor differs from propositional imagery in that it involves forming a particular mental image without necessarily involving any additional attitudes (this type of imaging is also known as ‘objectual imagining,’ see Yablo, 1993). For example, when you are imagining an ice-skating rink in Antwerp, you are not imagining *that there is an ice-skating rink in Antwerp*. Rather, you are forming a particular (in this case) visual mental image of a skating rink. Of course, not all cases of this type of mental imagery can meaningfully be characterized as picture-like experiences. Visual imagery tends to have a pictorial phenomenology (although see Thompson, 2007) but imagery associated with other modalities such as gustatory, auditory, tactile, or olfactory mental imagery do not. For example, when you imagine the smell of lavender, your experience is not picture-like since it does not involve having a visual experience of lavender (i.e., a visual mental image). Rather, it involves having an experience of its smell (i.e., an olfactory mental image) that lacks the phenomenal character of picture-like mental imagery^2^.

Mental imagery resembles perception in at least three ways. First, mental imagery and perception can have a similar phenomenology. This idea can be traced back to Hume (1739/1978, pp. 1–2) who argued that there is a “great resemblance” between mental imagery and perception “in every other particular, except their degree of force and vivacity” with the former being more “faint” than the latter. Imagining the smell of lavender and smelling lavender, for example, seem to have a similar phenomenology, although the former may not be as vivid as the latter^3^. Secondly, both mental imagery and perception bear *intentionality* insofar as both are about something, e.g., a president, a concert or a unicorn (Harman, 1998). Just as in the case of perception, the nature of the content of mental imagery will depend on, among other things, what the mental imagery is about (e.g., a scene, an object, a property). Thirdly, imagining something and perceiving something can occur actively and voluntarily as well as passively and involuntarily. For example, you may voluntarily recall ice-skating in Antwerp, because you have fond memories of ice-skating there with your friends. But you can also form a mental image involuntarily. You might, for example, have a flashback of a traumatic event you witnessed in your childhood. Or if hallucinations and dreams are kinds of mental imagery (Nanay, 2016b), you may passively hallucinate voices of powerful figures or passively dream that you won the essay competition you just submitted your paper to. Similarly, you can perceive something voluntarily, say, when you wish to attend to your child showing you some new moves at the ice-skating rink, but you can also perceive something even when you do not intend to perceive it, say, a horrific accident that is happening right in front of you.

There is, however, a crucial difference between mental imagery and perception. While mental imagery is the maintenance of a stable conscious representation in the absence of (relevant) sensory stimuli, perception occurs only when a stimulus is present (or at least a proximal stimulus in the case of hallucination). For example, you cannot have a (veridical) visual experience of an elephant in the absence of sensory stimulation^4^. Having a mental image of an elephant, by contrast, does not require that an elephant be present since mental imagery can be generated in the absence of sensory stimulation. Similarly, you cannot taste, hear, touch, or smell, something unless the relevant distal stimulus is present but you can imagine a sound, smell, or taste in the absence of sensory stimulation (Bensafi et al., 2012; Arshamian and Larsson, 2014). One explanation of this similarity between the phenomenology of perceptual experiences and that of episodes of mental imagery is that the two types of experiences represent the same determinable properties, e.g., red, quadrilateral, etc. (Nanay, 2015). Like peripheral vision or low-luminance pictures, visual imagery represents only rather abstract determinable properties (e.g., red, quadrilateral). Ordinary focal perception, on the other hand, usually represents these determinables by representing fairly determinate instances of the determinables (e.g., crimson, rectangular). For something to be crimson is for it to be red in a specific way. So, if an experience represents an object as instantiating crimson, it also represents that object as instantiating red. One possible explanation of this difference between perception and imagery is that the properties that imagery attributes to the scene are provided by other mental states (e.g., memory, expectation, beliefs), whereas the properties perception attributes to the scene are provided by (relevant) sensory stimulation (Nanay, 2015). Although this account may explain the very meager phenomenal content of visual imagery, it does not explain why visual imagery is not as glitzy, rich, and intense as a hand-on experience of, say, a beautifully colored mind-independent scene. A more plausible explanation for why mental imagery typically is fairly dull and lusterless in its phenomenal presence is that visual imagery is lacking in brightness and possibly also luminance contrast (for a review, see Brogaard, 2015). This is likely due to a lack of recruitment of early visual cortex in imagistic tasks as well as an inability to engage subcortical structures along the normal visual pathway.

The phenomenological similarity between imagery and perception suggests anatomo-functional overlaps. Studies on brain-damaged patients reporting imagery deficits that parallel perceptual impairments have given rise to the hypothesis that visual experience and visual imagery share mental operations and depend on common neural substrates (Farah, 1984; Levine et al., 1985; Damasio, 1989; Kosslyn, 1994). For example, studies suggesting that visual mental imagery (much like visual perception) depends on the functioning of retinotopically organized areas in the occipital lobe have led researchers to assign a crucial role to the primary visual cortex (V1) in mental imagery as well as to extra-striate visual areas such as V4, V5/MT and the fusiform area (Kosslyn et al., 2006).

It was observations along these lines that led Kosslyn (1981) to propose a Humean model of mental imagery according to which visual mental images are depictive, or “quasi-pictorial” representations (Kosslyn, 1994; Kosslyn and Ochsner, 1994)^5^. On this view, mental images are pictorial representations of objects or events, rather than symbolic, linguistic representations (or propositions) as suggested by Pylyshyn (1984) (see also Dennett, 1969, 1979; Pylyshyn, 1973, 1978)^6^. If mental images are pictorial rather than symbolic, linguistic representations, then the information they convey should be similar to the information conveyed by perceptual experiences, and the operations that can be performed on them should be similar to the operations implemented when a subject is perceiving the external world. For example, if it takes a certain amount of time to scan a real map, it should take, approximately, the same amount of time to scan a memorized mental image of the map. This latter mental-operations hypothesis has indeed been confirmed in multiple studies (see e.g., Shepard and Metzler, 1971; Kosslyn, 1975; Kosslyn et al., 1978; Shepard and Cooper, 1982; see also Tye, 1991).

As noted, Kosslyn’s view was in part motivated by the hypothesis that visual imagery and visual perception have a shared underlying mechanism, which would explain the informational and operational overlap between the imagistic and perceptual domains. Recent results from transcranial magnetic stimulation (TMS) studies indicate that adaptation to visual stimuli affects the ability to generate visual mental images, which suggests a functional overlap between visual perception and mental imagery in the early visual cortex (V1/V2) (Cattaneo et al., 2012). Other findings indicate an equivalence between perceptual and imagery processes that extends beyond V1/V2, involving interhemispheric exchange of information (Savazzi et al., 2008)^7^. As we will see below, however, this second component – i.e., the hypothesis that visual imagery and visual perception have a shared underlying mechanism – is not the only way to explain the core thesis of Kosslyn’s view.

## Kosslyn’s Imagery Model

Kosslyn’s model (as formalized by Farah, 1984) posits a single visual buffer which is used by both a bottom–up encoding system for visual perception and a top–down generation process for visual mental imagery. During object recognition, for example, an external image is projected to the retina by a bottom–up encoding system, which passes through a visual buffer as well as various stages of early visual processing in the parietal and temporal lobe leading to the activation of stored associative memories enabling its recognition. During visual mental imagery of an object, stored associative memories are activated by a top–down generation process and projected down the same visual pathways onto the same visual buffer used for object recognition. Afferent and efferent connections from one visual area to the next (Van Essen, 1985) as well as direct cortico–cortico connections from higher-level to lower-level visual areas are thought to facilitate this bi-directional flow of information (Douglas and Rockland, 1992; Behrmann et al., 1994).

As mentioned above, Kosslyn posited that area V1 is the most likely neural substrate for the visual buffer (Kosslyn, 1994). The evidence here seems to be inconclusive. Brain stimulation (TMS) studies indicate that the early visual cortex (V1/V2) plays a causal role both in the initial encoding of the visual input into working memory and the subsequent maintenance of a mental representation (Cattaneo et al., 2009). Although most models on working memory suggest that working memory representations are conscious by definition or directly accessible for conscious introspection, it has been suggested that the introspection of the information stored in working memory requires a new representation, which exists in parallel with the actual memory trace (see Jacobs and Silvanto, 2015). On this model, the content of working memory (which is used for conscious examination or manipulation) does not operate on the actual memory trace, but requires a new representation to be created (which is generated in addition and in parallel to the actual memory representation) for the conscious domain. It is possible, therefore, that the visual imagery and visual short-term memory share similar neural resources associated with the creation of distinct new representations.

Evidence from TMS studies furthermore indicates that the interhemispheric transfer time of phosphenes both when they are experienced and when they are imagined is slower when generated by V1, a delay which is attributed to its sparse callosal connections (Marzi et al., 2009). TMS studies of hemianopic patients (i.e., patients with an intact hemifield and a blind hemifield often resulting from a stroke) also indicate that the activity of the occipital cortex is not a constituent of the biological basis of the experience of phosphenes, suggesting that such experiences can be generated independently of any contribution from V1 (Mazzi et al., 2014; Bagattini et al., 2015). It should be pointed out, however, that since the experience of phosphenes differs from visual perception in that the former experience is not attributable to an external stimulus, it is not clear whether these findings are sufficiently informative in terms of the relation between visual perception (which involves experiences of external stimuli) and mental imagery. Perhaps, the observed similarities can be attributed to the fact that visual experiences of phosphenes and mental imagery arise in the absence of an external stimulus, where V1 plays a central role in computing brightness perception (see the section below titled “What Explains the Phenomenology of Mental Imagery?”). If this is indeed the case, then the similarities between experiences of phosphenes and imaginations is not a good indicator of the nature of the relation between visual perception and mental imagery.

The current evidence from brain lesion studies is inconclusive. For example, a study comparing normal participants with a hemianopic patient testing reaction times for peripheral versus central stimuli (which is typically slower for the former than the latte) found a similar retinal eccentricity effect in normal participants but not in the hemianopic patient who had no difficulty imagining stimuli (Marzi et al., 2006). These results indicate that deafferentation of the visual cortex disrupts the visuotopic organization shared by visual perception and mental imagery. Many brain lesion studies discussed in the introduction, however, indicate that lesions in the occipital lobe do not typically produce deficits of visual mental imagery and that visual mental imagery deficits can occur even when V1 remains intact (Bridge et al., 2012; de Gelder et al., 2015). These findings suggest that occipital damage may not be sufficient for visual imagery deficits (Bartolomeo, 2002; Moro et al., 2008). Perhaps visual mental imagery is based on neural representations that are similar to those involved in visual perception, but that the former involves high-level representations, for example, shared object category representations (e.g., “dog,” “tree,” “table,” etc.) in category-selective regions on the ventral (and less so on the lateral) cortical surface (Ishai et al., 2000; O’Craven and Kanwisher, 2000; Cichy et al., 2012). Indeed, the available evidence suggests that brain areas related to attention, memory retrieval, motor preparation, semantic processing, default-mode network, and multisensory integration subserve supramodal imagery processes for visual as well as auditory information (Zvyagintsev et al., 2013)^8^. This also seems to be the case for olfactory mental imagery. While studies show that olfactory mental imagery arises from neural activity in early olfactory cortices, i.e., the piriform cortex (Djordjevic et al., 2005; Bensafi et al., 2007)^9^, large individual differences in the capacity to reproduce olfactory conscious images (Arshamian and Larsson, 2014) suggest that olfactory mental imagery relies on the activity of high-level representations such as attention, expectation, and memory (Bensafi et al., 2012; Royet et al., 2013). The diminished capacity to reproduce olfactory conscious images could, therefore, be explained in terms of memory deficits: if we are less likely to remember olfactory information, we will also be less likely to replicate it in imagination.

Findings showing dissociation across domains, however, suggest that visual (as well as tactile) imagery deficits can occur independently of deficits of visual (or tactile) perception (Basso et al., 1980; Farah et al., 1988; Riddoch, 1990; Moro et al., 2008). For example, a study on a patient with bilateral lesions to extrastriate visual areas found that the ability to vividly imagine objects, letters, colors, and faces was intact despite severe perceptual impairment in object (object agnosia), letter (pure alexia), color (achromatopsia), and face recognition (prosopagnosia) (Bartolomeo et al., 1998). In another study, two cortical blindness patients with bilateral medial occipital damage retained their ability to imagine object forms (Chatterjee and Southwood, 1995). The opposite dissociation, that is, impaired visual mental imagery with fairly intact perception, was also found in two patients with brain damage from closed head injury, with one of them also showing tactile deficits (Moro et al., 2008). In both patients the left temporal lobe was impaired but there was no visible damage to the occipital cortex, which suggests that the temporal lobe is crucially involved in visual imagery. Studies also indicate that patients with normal color vision can have impaired color imagery (De Vreese, 1991; Luzzatti and Davidoff, 1994). For example, Luzzatti and Davidoff (1994) presented two patients (GG and AV) with a picture naming task which included 12 natural objects such as fruit or vegetables and 32 artificial objects. Both showed impairments of color imagery. However, GG was found to have slightly greater impairments of color imagery with natural objects compared to artificial objects. Indeed, the current evidence from brain lesion studies suggests that a closer correspondence seems to exist for motor imagery and motor action than for visual imagery and visual perception (Sirigu et al., 1996; Bartolomeo et al., 2012; for an account of motor imagery see Annett, 1996).

Although the overall evidence on whether visual perception and mental imagery share similar mechanisms is inconclusive, there seems to be a considerable overlap between the domains of vision for action and imagined perspective taken, motion and rotation. In what follows, we argue that this provides support for a modification of Kosslyn’s mental imagery model.

## Vision for Action: Rethinking Kosslyn’s Model of Visual Imagery

As we suggested above, the current evidence as to whether Kosslyn’s quasi-pictorial account of visual imagery is viable is inconclusive. More importantly, they are focused almost exclusively on conscious imagistic acts (see also Abell and Currie, 1999). However, even if the evidence were to definitely suggest that there is no significant overlap between vision for perception and conscious imagery, that would be insufficient to undermine a pictorial model of (visual) imagery. Different mechanisms and neural substrates can give rise to the same or similar experiences. For example, in blind people who navigate the world using human echolocation, the echos give rise to a distinctly visual phenomenology (Thaler et al., 2011). The fact that different mechanisms underlie vision for perception and conscious visual imagery, therefore, does not preclude there being a significant overlap in phenomenology (although see Kriegel, 2015). So, to the extent that the phenomenology of visual perception is pictorial, the phenomenology of visual imagination may well be pictorial too.

Kosslyn’s model, however, was not simply a hypothesis about the phenomenology of imagery but also about the mechanism and neural substrates that support it. Although Kosslyn was primarily concerned with conscious imagery, a case could be made for a significant overlap between the mechanisms underlying vision for action and unconscious visual imagery^10^. Vision for action is the visual processing required to retrieve information about the features of objects needed to guide online movement such as manipulating a computer mouse, quickly reaching to and grasping an object, or jumping from one rock at the bay to another rock located some distance away. The visual processing required to retrieve this sort of information has been shown to be functionally and anatomically distinct from vision for perception – the processing required to recognize an object or its attributes (Goodale et al., 1991; Goodale and Milner, 1992; Milner and Goodale, 1995, 2008; although see van Polanen and Davare, 2015). David Milner and Melvyn Goodale argued that the two types of vision correspond to two functionally specialized cortical streams of visual processing originating in the primary visual cortex (V1): a dorsal, action-related “unconscious” stream and a ventral, perception-related “conscious” stream. The dorsal stream computes information about absolute size and orientation and viewpoint-dependent properties of objects in egocentric space whereas the ventral stream processes information about color and shape and relational properties of objects in allocentric (scene-based) space (Schenk, 2006). Whereas the dorsal stream normally operates in the absence of visual awareness, ventral stream processes often correlate with visual awareness.

The initial evidence for this dissociation hypothesis came from studies on brain-lesioned patients resulting in visual agnosia, which can affect not only the ability to recognize objects and faces but also the ability to create visual images of objects and faces (Charcot, 1889; Goldstein and Gelb, 1918; Spalding and Zangwill, 1950; Brain, 1954; Macrae and Trolle, 1996; Farah, 2004; Brogaard, 2011a,b). Milner and Goodale’s original studies indicated that lesions to the dorsal stream can negatively affect visuomotor control even when visual perception is largely intact. Similarly, ventral stream damage can negatively affect visual perception without impairing visuomotor control (see also Farah, 2004). These results were based on several neuropsychological studies that were carried out on a patient, D.F., who had visual form agnosia following CO poisoning with accompanying damage to the ventral stream (see Goodale et al., 1991; Goodale and Milner, 1992). While D.F. was visually aware of some texture and color, her ability to visually detect objects and shapes was impaired. Her dorsal stream was unaffected by the accident. Even though she was unable to describe objects, she was able to extend her hand to the location of an object and grasp it. For example, she was able to place a card into a mail slot and modified her grip aperture accurately to the size of a rectangular block. When there was a delay in her actions, which allowed her to rely in part on the ventral stream, however, this led to an impairment in performance (McIntosh et al., 2004).

Because D.F. was unable to consciously identify shapes and objects maintain working memory of visually seen objects but was able to reach to and grasp these objects, Milner and Goodale argued that the information processed in the dorsal action-guiding stream is not stored in working memory. When actions involve keeping a visual image in working memory, the ventral stream is recruited.

Milner and Goodale’s hypothesis to the effect that the ventral and dorsal stream are functionally and anatomically dissociated was also studied in patients with optic ataxia. Optic ataxia patients are unable adjust their hand aperture to the size of objects unless the action is delayed. Milner et al. (2001) found that optic ataxic patient I.G., who had damages to the posterior parietal cortex, had difficulties adjusting grip aperture to object size when the task was to grasp the object immediately upon seeing it. When the action was delayed, an improvement was observed between I.G’s grip aperture and the size of the object. This indicated that I.G’s ventral stream controlled memory-guided action. Additional evidence indicated that when the object remained visible, I.G. was able to use information associated with the ventral stream.

Since the double dissociation hypothesis was first proposed, there has been significant debate about the extent to which vision for action really is unconscious and whether there is significant interaction between the ventral “vision-for-perception” stream and the dorsal “vision-for-action” stream (for a review, see Brogaard, 2011a). The evidence overwhelmingly suggests that vision for action proceeds largely via processes that are inaccessible to consciousness, for instance, processes that underlie estimations of the distance from oneself to a target object, the size of the object, the route from oneself to the object and adjustments of body parts prior to the movement-related tasks (e.g., adjustment of hand aperture) (Brogaard, 2011a).

Vision for action is considered a kind of vision because it requires visually estimating absolute object size, weight and orientation and perspectival properties of entities located in egocentric space (Schenk, 2006). However, when we look closer at the mechanism underlying vision for action, it becomes evident that vision alone cannot account for the dorsal-stream representation generated in preparation for online action. Processing associated with vision for action involves multi-modal integration (Gentilucci et al., 1995; Jacob and Jeannerod, 2003; Schenk, 2012; although see also Milner et al., 2012; Whitwell and Buckingham, 2013). In spite of the fact that vision is the central component in vision for action, the content of dorsal stream representations consists of various non-visual stimuli, including haptic, kinesthetic, or proprioceptive. Subjects adjust their grip size to match the object, even when changes in the size of an object are not consciously detected (see Gentilucci et al., 1995). Haptic and proprioceptive cues from the hand indicating the size of the object aid in the adjustment of kinematic movements associated with reaching and grasping it. Action-guiding representations also involve imaginings of the route that needs to be traveled to bypass obstacles and reach an object or location (cf. Jeannerod, 1994). So if action-guiding representations in the dorsal stream are largely inaccessible to consciousness, the relevant imaginings would also seem to be largely outside the reach of consciousness.

The current empirical evidence supports the hypothesis that the imaginative processes needed for generating multimodal action-guiding representations are unconscious (Paulignan et al., 1991; MacKenzie and Iberall, 1994, Chap. 5). In order to compute reaching- and grasping-behavior, the brain relies on visual representations of the object and the location of the object, proprioceptive representations of hand and arm as well as an estimation of the route from the hand and arm to the object. Estimating the path to the object requires imagery. Yet when the action is online (i.e., fast and ongoing), this imaginative process is not conscious^11^. If an object abruptly jump to a new location, the velocity and trajectory of the arm can be carried out in less than 100 ms, which is insufficient time for the brain to generate conscious representations of the change in the object’s position or the alterations in the hand’s speed or trajectory (Paulignan et al., 1991). It has been found that when research participants are requested to utilize a vocal sound (Tah!), which is minimally cognitively demanding, to indicate whether they were conscious of a change in the location of the object, they adjusted their movements long before they were able to utter the sound ‘Tah!’ Whereas the vocal response took place after 420 ms, corrections of trajectory and grip aperture occurred within 100 ms, which means that the new route must have been imagined in less than 100 ms (Castiello and Jeannerod, 1991; Castiello et al., 1991).

The current evidence from studies pertaining to pointing and saccadic eye movement indicate that subjects can modify pointing and saccadic eye movements more rapidly than they can consciously perceive a change in the location of an object (Goodale et al., 1986; Pelisson et al., 1986). For example, subjects in one study were instructed to point as quickly and as accurately as possible to targets viewed in the dark (Pelisson et al., 1986). The experiment included two series of trials. In the first, the target made only one movement: it jumped from an initial position to a randomly selected position. In the second, the target made two movements: after jumping to a randomly selected position, it jumped back to same initial position. Although pointing and saccadic eye movements were immediately modified to match the second location of the target, subjects reported that they were not aware of the location of the target’s second jump and were unable to predict its location. In spite of the fact that the subjects were not consciously aware of the two jumps, they were evidently unconsciously seeing both jumps and were imagining a new path for the target. The study suggests that the participants adjusted the trajectory of their movements without being aware of the adjustments. Hence, their imaginings of the movement trajectory and the target location proceeded below the level of conscious awareness.

In another study, Jakobson and Goodale (1989) reported similar findings. While uninformed subjects failed to detect (small) prism displacements (five diopters), their modifications to the reach trajectory suggested that they accurately adapted to the prism’s distortions. This suggests that the subjects were unconsciously making corrections. It has also been found that while subjects are not aware that they rely on visual information about their hand and its impending path prior to the motion of the hand, their performance has a higher degree of accuracy when the subjects do have access to this information (Prablanc et al., 1979; Elliott et al., 1991; Rossetti et al., 1994; Desmurget et al., 1995).

It may be argued, of course, that predicting a path of movement or a target location is not a form of imagining. Imagining, it may be said, is essentially an activity governed by the ventral stream rather than the dorsal stream. Evidence, however, does not support this hypothesis. Although spatial neglect typically involves a failure to use one side of the body or recognize entities on one side of the body, it has been found that some subjects with neglect fail to be able to visualize familiar spatial locations (Guariglia et al., 1993). A plausible explanation of this finding is that an intact body schema is required for visual imagery of spatial locations, because it requires an ability to locate oneself as an acting force in relation to the spatial location. As Jeannerod (1994, p. 191) puts it, “representation of the self in movement... requires a representation of the body as the generator of acting forces, and not only of the effects of these forces on the external world” (see also Coslett, 1998). Given that the body schema is an integral part of vision for action, imagery is not restricted to the ventral stream. This further suggests that the processes of determining a movement path (or a route to a target) and a target location are imaginative processes that fail to reach conscious awareness.

Unconscious imagery thus appears to be an integral part of vision for action, suggesting a partially shared mechanism for these two domains. The question is to what extent an overlap between unconscious visual imagery and vision for action can support Kosslyn’s imagery hypothesis. The theory of mental imagery originally presented by Kosslyn (1981, p. 47) was intended to provide an account the processing involved in “‘looking’ at images, and of transforming images in various ways.” This process of moving around in one’s own imagery or transforming images seems to involve movement as well as an anticipation of movement that is similar to the anticipation of movement represented by dorsal stream action-guiding representations. In the cases Kosslyn describes, there is evidently also a conscious element. When we are asked to count the number of windows in a room, for example, there is a conscious representation of parts of the room. But the scanning over the walls to find the windows requires mentally positioning ourselves at a certain distance from the walls and in some cases ‘moving around’ furniture and other obstacles. This activity seems to involve processes below the level of conscious awareness. For example, estimating the length we need to travel to get from one window to the next within the image involves subpersonal processes. So, one element of Kosslyn’s original hypothesis can be preserved: visual imagery is at least partially grounded in a neural substrate and mechanism utilized by vision, albeit vision for action rather than vision for perception. This hypothesis does not explain other crucial parts of the model. For example, it does not explain why we should believe that visual imagery is quasi-pictorial. We turn to this question next.

## Does the Quasi-Visual Phenomenology of Conscious Imagery Support an Enactive Theory?

After reviewing evidence in support of the view that visual and imaginative processing are functionally and anatomically distinct processes, Bartolomeo (2002) attempts to answer the question of where the ‘quasi-visual’ phenomenal character of visual mental images come from. His answer relies in part on the so-called enactive view of perception defended by O’Regan and Noë (2001) and others (for a book-length defense see Noë, 2004). On the enactive view, perceptual experience cannot be understood as an internal representation of the perceived environment, but must be understood in terms of knowledge of the actions involved in perceiving the environment. Seeing an object, for example, involves a kind of knowledge of all the ways the shape changes when we move around it or it moves relative to us. For example, if we turn a coin in our hands, the coin’s circular shape will turn into an oval shape. On the enactive view, the phenomenology of visual experience is constituted by the exercise of our knowledge of these kinds of sensorimotor contingencies.

Bartolomeo (2002) suggests a way to understand the ‘quasi-visual’ phenomenal character of visual mental images within the enactive framework. The ‘quasi-visual’ phenomenal character of mental imagery could be understood as partly constituted by an exercise of the knowledge of the sensorimotor contingencies that also constitutes perception on the enactive view. However, the phenomenology of imagery is impoverished compared to visual experience as a result of the absence of an external stimulus. The difference between visual experience and visual imagery, on this view, is that visual experience is constrained by the external environment, whereas visual imagery is constrained by memory processes. The constraining factors may explain the different neural correlates underlying the two domains.

Bartolomeo’s proposal is disputable on the grounds that it implies that vivid hallucinatory experiences are at least partly constituted by an exercise of knowledge of sensorimotor contingencies, and, like imagery, the hallucinatory aspects of the experience fail to be constrained by the external environment. Yet hallucinatory experience can have a phenomenology that is just as vivid and pictorial as perceptual experience (Brogaard, 2013; Brogaard and Gatzia, 2016). This undermines Bartolomeo’s proposal as an explanation of the pictorial phenomenology of visual imagery, which is normally fairly impoverished compared to hallucinations. A further problem with Bartolomeo’s suggestion is that active imagery often involves what William James referred to as a ‘sense of effort’ (James, 1892). This is the feeling that one is the agent of the imagistic activity. This sense of effort is what distinguishes imagery from hallucination and to some extent also veridical perceptual experience.

Moreover, there is an independent challenge facing the enactive approach to perception and visual imagery. As Block (2005) has argued, on the assumption that sensorimotor know-how is a kind of know-how of visually-guided action, the enactive dogma cannot easily account for the phenomenology of visual experience. Block points to a study by Goodale and Murphy (1997) who demonstrated that subjects can effortlessly reach to an object and grasp it even when it appears blurry. In the cited research, five rectangularly-shaped blocks were arranged in various locations (ranging from 5 to 70 off the line of sight) in the subjects’ visual field. At 70, subjects visually represented the blocks blurrily and found it hard to distinguish them but had no difficulty reaching to and grasping the blocks. Interestingly, the difference between the participants’ grasping performance at 5 and 70 were not statistically significant. This demonstrates that we can grasp objects even when they are hardly visible. These findings indicate that whatever lies beneath our representations of the online action we are about to perform cannot be what lies beneath perceptual experience, because representations of anticipated online action are largely unconscious, whereas perceptual experience by definition is conscious. For example, one study that looked at the mental representations underlying motor imagery and corresponding action in a subject (CW) with lesions to bilateral parietal areas found that when imagining movements of his hands, CW executed the imagined movements in the absence of conscious awareness (Schwoebel et al., 2002).

**FIGURE 1:**
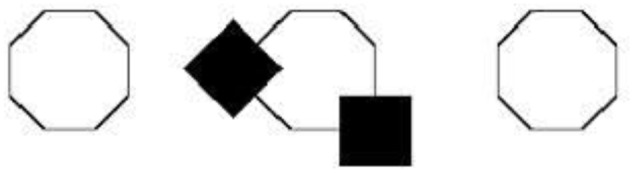
**Kanizsa amodal completion.** Although the flanking octagons should increase the likelihood of the occluded figure in the middle being a regular octagon, the occluded figure is not seen as such Pylyshyn (1999).

The argument in the previous section provides further evidence against the enactive view, whether construed as an account of perception or imagery. O’Regan and Noë may indeed be right that sensorimotor know-how is required in order for perceptual experience to take place. Likewise, Bartolomeo may be right that sensorimotor know-how is also required in order for imaginative experience to occur. Neither observation, however, gives us good reason to think that sensorimotor know-how is constitutive of (or explanatory of) the phenomenology of perception or imagery. Since the dorsal-stream representations underlying both domains are inaccessible to consciousness, they can at best be part of the perceptual or imaginative mechanisms responsible for generating conscious perceptual or imaginative experiences. Just like the processes taking place in LGN or the primary visual cortex, which ultimately lead to a conscious experience, are not constitutive of the phenomenology of the experience, so sensorimotor know-how need not be constitutive of the phenomenology of experience. We can compare these dorsal-stream processes to the intra-perceptual principles or ‘organizing principles of vision,’ that modulate early visual processes (Fodor, 1983; Pylyshyn, 1999; Raftopoulos, 2001; Brogaard and Gatzia, 2017). For example, in the case of amodal completion, partially occluded figures such as the polygon in the middle in **Figure 1** are not perceived as the fragments of the foregrounded figures. Rather, they are perceived as concealed or masked by the occluder. Visual processes seem to be modulated by intra-perceptual principles, which facilitate the completion of the concealed parts of the occluded figures (**Figure 1**).

These intra-perceptual principles are not consciously accessible rational principles (e.g., maximum likelihood or semantic coherence)^12^. The visual system employs them to compensate for the inherent ambiguity of proximal stimuli. In **Figure 1**, the presence of the outermost regular octagons should increase the likelihood that the occluded figure is also a regular octagon. But the principles of amodal completion are executed on the basis of their own algorithms, and the occluded figure is not experienced as a regular octagon.

Intra-perceptual principles work below the level of conscious awareness and are likely inaccessible to consciousness even in principle, so they occur at a subpersonal level. Accordingly, they are not constitutive components of perceptual experience. Likewise, unconscious dorsal-stream processes, or what O’Regan and Noë call ‘sensorimotor know-how,’ are not accessible to consciousness and so are not constitutive components of conscious imagery or conscious perception. So Bartolomeo’s (2002) suggestion that the dissociation between the visual and imaginative domains support an enactive approach to the mind is erroneous, at least if the enactive approach is proposed as a theory about the constitution of conscious mental states.

## What Explains the Phenomenology of Conscious Imagery?

If conscious imagery is indeed quasi-pictorial but fails to have the same neural substrate as perception, what explains its vivid perception-like phenomenology? It is commonly accepted that the encoding and retrieval of episodic (imagistic) memory and the maintenance of (imagistic) working memory consists in a reinstatement of activity in the neural circuits that originally processed the perceptual stimuli (Gazzaley et al., 2004; Postle, 2006; D’Esposito, 2007; Fuster, 2009; Serences et al., 2009; Danker and Anderson, 2010; Rissman and Wagner, 2012). The information from the various neural circuits are then integrated to form the memory representation. The reinstatement hypothesis extends to other forms of imagery such as daydreaming and imagination that integrate memory fragments in novel ways (Lyons, 1986; Kosslyn, 1994; Hassabis et al., 2007; Pearson et al., 2015).

Initial appearances to the contrary, the reinstatement hypothesis is indeed consistent with the double dissociation between the visual and the imagistic domains. As noted above, whereas perception proceeds primarily via bottom–up processing, imagery proceeds primarily via top–down processing. It is to be expected, therefore, that dissociations of bottom–up and top–down processing in a single visual system that governs both mental imagery and perception can occur (Shuren et al., 1996). Consider double dissociation between color perception and color imagery. There are reports of individuals with color vision but no color imagery (De Vreese, 1991). Conversely, cases have been described in which patients with achromatopsia cannot perceive color but are nonetheless able to visualize color (Shuren et al., 1996). If a lesion in the V4/V8 cortical area affects only bottom–up processing but not top–down reactivation, we should expect cortical color blindness but not an absence of color imagery. If a lesion to the system impacts top–down re-activation but not bottom–up processing, on the other hand, then color vision may be preserved but color imagery will be impaired. This represents a limitation of double dissociation studies as a cornerstone in arguments against the pictorial model of mental images. These studies do not necessarily show that there is no overlap of neural substrates but only that there is not a full overlap of mechanisms.

What explains the pictorial phenomenology of visual imagery, then, is that it is processed in visual systems that also process matching visual experience. This raises the question of why the phenomenology is impoverished. Although some evidence seems to suggest that the primary visual cortex is crucial in both visual perception and conscious imagination (see Cattaneo et al., 2009, 2012), one explanation may turn on the difference in the involvement of the primary visual cortex in visual perception and conscious versus unconscious visual imagery. Blindsight studies have shed light on the importance of the primary visual cortex (V1) in processing brightness (awareness of luminance). Blindsight is the result of lesions to V1 which give rise to a region of blindness (a scotoma) in the visual field (Poppel et al., 1973; Weiskrantz et al., 1974; Perenin and Jeannerod, 1975). Subjects with this condition do not acknowledge being aware of visual stimuli that are shown in their blind hemifield. They are, however, capable of making correct guesses about features of visual stimuli shown to them when they are forced to guess what is in front of their eyes. Studies have shown that blindsight subjects tend to make above-chance discriminations of various features, including their wavelength, location, motion, and form, of visual stimuli they report being visually unaware of Weiskrantz (1986) and Stoerig and Cowey (1992).

Blindsight was originally considered to be the possession of residual visual abilities in the absence of acknowledged visual awareness. However, recent findings indicate that some blindsight subjects have residual conscious awareness in their affected hemifield in spite of extensive V1 lesions. Nevertheless, these subjects are still considered blindsight subjects because they have residual vision for stimuli features they are not aware of. Subjects have reported residual awareness of the presence and direction of fast moving and/or high-contrast stimuli. There is often a positive correlation between such residual awareness and the abilities of these subjects to make above-chance discriminations (see Barbur et al., 1993; Zeki and Ffytche, 1998).

A division of blindsight into types 1 and 2 has resulted from the observation that some blindsight patients have residual visual awareness (Weiskrantz, 1998a,b). In type 1 blindsight, subjects with lesions to the primary visual cortex have the ability to detect object attributes in spite of being unaware of them – studies suggest that in differentiating between genuine forms of type 1 blindsight and degraded conscious vision graded measures for assessing awareness are better than “guest” trials (see Mazzi et al., 2016). In type 2 blindsight subjects with damage in the primary visual cortex (V1) have some residual visual awareness, although they are unaware of most of the features of objects presented to them. Some patients have reported conscious visual awareness of the motion of objects or knowledge that something had moved through their blind hemifield but these subjects deny that they could see the shape or color of the moving object, or when the object is described as having a color, it is typically said to be ‘shadowy gray’ or ‘like a shadow’ (Zeki and Ffytche, 1998).

Verbal reports clearly indicate that the phenomenology of type 2 blindsight and normal visual experience is radically different (Stoerig and Barth, 2001; Weiskrantz, 2009; Ffytche and Zeki, 2011). Stoerig and Barth (2001) conducted a study that aimed at finding a visual stimulus that, when presented to GY’s sighted hemifield, would be phenomenologically akin to how he saw objects presented to his blind hemifield. Stimuli with reduced spatial and temporal resolution were initially believed to be able to trigger visual experiences on a par with GY’s type 2 experience but GY judged them to be dissimilar. To trigger a reasonable match the researchers needed to present a moving low-contrast texture to the sighted hemifield and a moving luminance-defined bar to the blind hemifield. The fact that dissimilar stimuli had to be presented to the sighted and the blind hemifields to ensure a match strongly indicates that different attributes of the stimulus enter the brain from the sighted and the blind hemifields or that the brain processes the same attributes differently. This suggests that the phenomenology of normal visual experience and experience in type 2 blindsight are fundamentally different.

The reason type 2 blindsight is categorically different from ordinary visual experience is likely that type 2 blindsight is the result of processing in an atypical visual pathway that bypasses the primary visual cortex (V1). A examination of GY’s abilities with respect to matching luminance in his blind hemifield and between both hemifields indicated that GY was unable to make the matches when a stimulus was presented only in his blind hemifield but could make matches it was presented in opposite fields (Morland et al., 1999). The most likely explanation for this results is that the perceived luminance of a stimuli in GY’s blind field (i.e., the perception of brightness) arises from direct projections from subcortical areas to extrastriate areas, which bypass the primary visual cortex (V1). The perceived luminance of a stimulus in his intact field, by contrast, seems to originate in the normal visual pathway which includes V1. This would enable GY to compare stimuli that are presented in the opposite fields. When the stimuli are presented to opposite fields, however, the distinct pathways would yield different kinds of percepts making matching difficult. These findings suggest that the primary visual cortex (V1) plays a central role in computing brightness perception. The reason type 2 blindsight has a radically impoverished phenomenology compared to ordinary visual experience may thus be that it is accompanied by a loss of luminance awareness (Brogaard, 2015). It is likely that the loss in luminance awareness in type 2 blindsight emerges in a visual pathway that bypasses the primary visual cortex (V1) (see also Azzopardi and Hock, 2011).

It is likely that visual imagery has an impoverished phenomenology for much the same reason that type 2 blindsight does. Reinstatement of activity in V1 is not likely to be significant in coarse-grained visual imagery (Mellet et al., 1995, 1996; Roland and Gulyas, 1995; D’Esposito et al., 1997; Knauff et al., 2000). Although V1 is recruited, it appears to be recruited only when the imagery involves a representation of fine-grained spatial detail, for example, recalling whether a dog’s ears are pointy or floppy (Pearson et al., 2015). So, the lack of perception-like vividness of visual imagery may be due to a lack of sufficient processing of brightness in V1, which can lead to impoverished phenomenal content (Brogaard, 2015). This idea is consistent with the finding that the vividness of visual imagery seems to be strongly correlated with amount of activity in the early visual areas (Pearson et al., 2015). An additional factor that may influence the vividness of visual imagery is absence of bottom–up processing in subcortical structures in visual imagery. Visual experience is generated on the basis of cortical processing of information that is already processed in the subcortical structures (e.g., SC and LGN) of the brain that project to the primary visual cortex (Schneider and Kastner, 2005). For example, the superior colliculus computes luminance contrast along with other features of the stimulus. The absence of this type of processing prior to the processing of chromatic contrast and hue in striate and extrastriate areas may be an additional factor contributing to the impoverished phenomenal content of visual imagery.

## Concluding Remarks

We have argued that although visual imagery is quasi-pictorial, it need not share the neural substrate or mechanism for vision for perception but likely does overlap with the neural substrate and mechanism for vision for action. This preserves a version of Kosslyn’s imagery model, one that postulates that vision and imagery have overlapping neural substrates and mechanisms that enable mental rotation and mental scanning processes that resemble their real counterparts.

These observations, however, do not explain why the conscious aspects of visual imagery are quasi-pictorial. We have argued that the pictorial phenomenology of visual imagery can be explained by the fact that it is processed in visual systems that also process matching visual experience. However, the mechanisms underlying conscious visual imagery and perceptual experience may be different, which would explain findings of double dissociation between perception and imagery even when a single visual system is involved (e.g., the V4/V8 color system). The impoverished phenomenology of mental visual imagery can, in turn, be explained by differences in the involvement of the primary visual cortex (V1). This receives support from the phenomenon of blindsight, which results from lesions to the primary visual cortex (V1) that lead to a region of blindness (scotoma) in the visual field but residual visual abilities and sometimes residual awareness. Contrary evidence indicating that, in normal (non-blindsight) cases, perceived and imagined stimuli exert similar effects on visual reaction time, e.g., an increase in luminance, contrast and visual motion decreased reaction time while gratings of low spatial frequency increased reaction time (Broggin et al., 2012), may be explained by the fact that V1 neurons respond to luminance and contrast changes. So even if there is no significant overlap between visual perception and mental imagery (a claim that is not fully supported by the overall evidence), there are compelling reasons for thinking that Kosslyn’s imagery model can be preserved. Namely, it is likely that the pictorial model (e.g., measurements of mental movement from one location to another) involves unconscious dorsal-stream representations – representations that are an integral parts of vision for action.

It should be noted that the pictorial view, as we envisage it, does not imply that conscious visual imagery is wholly constituted by representations with a quasi-pictorial phenomenology (cf. Peacocke, 1985; White, 1990; Kind, 2001; Wiltsher, 2016). Consider someone who imagines Donald Trump speaking at a Republican convention but whose mental image has pictorial phenomenology that would have been the phenomenology of a veridical experience or veridical memory of a middle-to old-aged Biff Howard Tannen from the Back to the Future trilogy. The quasi-pictorial phenomenology of the imagery, in this case, does not fully determine the imagistic content, that is, what the imagination represents. The agent’s beliefs about what Donald Trump looks like partially determines the imagistic content. Despite the fact that the mental image resembles an image of Biff Howard Tannen, the imagination represents Donald Trump. Here is another case. Consider someone imagining President Obama worrying about the financial crisis in the Oval Office in 2009. The pictorial phenomenology represents a worried face but not Obama worrying about the financial crisis. The additional content representing Obama’s mental state is supplied by the content of the agent’s thoughts about Obama’s mental states. So, the quasi-pictorial phenomenology of visual imagery does not fully determine the content of the imagination. In some cases what is represented by the qualitative features of the visual image is partially determined by the subject’s beliefs or suppositions, which is to say, that the imagination can represent content over and above what is represented by the visual image.

## Author Contributions

All authors listed, have made substantial, direct and intellectual contribution to the work, and approved it for publication. The order of the authors’ names appear in alphabetical order.

## Conflict of Interest Statement

The authors declare that the research was conducted in the absence of any commercial or financial relationships that could be construed as a potential conflict of interest.

## Footnotes

1 We use the term ‘mental imagery’ to refer to both re-experiences of an original stimulus as well as imagination. Although our conclusions can be generalized to other forms of mental imagery, the main focus of this paper is visual mental imagery.

2 Presumably, imagining the smell of lavender reactivates the limbic system rather than the cortical system. For discussions on olfactory, gustatory, and auditory mental imagery see Paivio and Csapo, 1969; Bensafi et al., 2012; Arshamian and Larsson, 2014.

3 Kriegel (2015) argues that the phenomenology of perception and imagery differs not just in vividness but also in kind, i.e., there is an attitudinal difference between the two (see also Wiltsher, 2016). This would be analogous to the difference in the phenomenology of belief and desire, even when we assume that the content is the same. One could argue, however, that the attitudinal difference between perception and imagery is not reflected in the phenomenology of the states but is reflected in the manner in which perception and imagery represent the scene (perceptually versus imagistically).

4 Or to put it more succinctly: you cannot have an accurate experience of an elephant in the absence of a distal sensory stimulus. You could, of course, hallucinate an elephant and happen by sheer coincidence to be standing in front of one. This would make your experience veridical (this is also known as a ‘veridical hallucination’). But in the envisaged scenario, the causal history of your experience would be mistaken; hence, your experience would not be accurate or successful.

5 The term ‘pictorial’ is applicable only to the phenomenology of visual imagery and not auditory, tactile, olfactory, gustatory or multisensory imagery. In what follows, we make this assumption without necessarily making it explicit.

6 It should be noted that Pylyshyn does not deny that there can be quasi-pictorial phenomenology associated with visual image but only that manipulating the mental images (e.g., in rotation and scanning tasks) takes the form of manipulating symbolic linguistic representations. An alternative to Kosslyn’s view of ‘mental pictures’ and Pylyshyn’s symbolic, linguistic representations takes mental images to represent the layout of perceived space, in Gibson’s sense (see Neisser and Kerr, 1973).

7 The current evidence suggests that visual callosal connections share a majority of anatomical and functional features with lateral connections less with feedback connections than on the same hierarchical level (see Schmidt, 2013).

8 Interestingly, a study found that visual imagery resulted in relative deactivation within the modality-specific areas for auditory imagery and vice versa while visual and auditory mental imagery significantly decreased activation in primary sensory and motor areas (Zvyagintsev et al., 2013).

9 Olfactory mental imagery was found to increase activation in the primary (i.e., piriform cortex) and secondary (i.e., insula and orbitofrontal cortex) olfactory areas (Djordjevic et al., 2005), with unpleasant imaginary and real odors inducing greater activity in these areas than pleasant ones (Bensafi et al., 2007).

10 *Conscious* imagination is likely to play a significant role in action preparation when action is delayed (for instance in cases of decision making), see, e.g., Currie (2002), Nanay (2016a), and Van Leeuwen (2016). Here, is an example from Nanay (2016a, 134): “When you decide between the two jobs, you imagine yourself in the situation that you imagine to be the outcome of your decision one way or the other. You imagine yourself at the prestigious university surrounded by great colleagues and doing excellent research in a sleepy small town, spending the evenings working or with colleagues. You also imagine yourself at the not so prestigious university, spending every night out in cool restaurants and at various cultural events, to return to teaching the next day among your mediocre colleagues and not-so-bright students. Then you compare these two imaginative episodes, and the one you prefer will be the course of action to follow.”

11 Similarly, in amodal perception, the imaginative process that fills in the missing parts of a stimulus is not conscious (see **Figure 1**).

12 These principles are akin to what Helmholtz called “unconscious inferences” (Gordon, 2004), what Gregory (2009) calls “hypotheses,” or what Bayesians call “implicit assumptions” (Rescorla, 2015). They are principles regulating processes that occur at a subpersonal level.
